# Vesico-Vaginal Fistula—continued

**Published:** 1867-11

**Authors:** C. C. F. Gay

**Affiliations:** One of the Surgeons of the Buffalo General Hospital


ART. III.—*Vesico-Vaginal Fistula—continued.* By C. C. F. Gay, M. D., *one of the Surgeons of the Buffalo General Hospital.*

The question has often been asked me which anæsthetic, chloroform or ether, is the more preferable for use in the operation for vesico-vaginal fistula? I unhesitatingly answer, the former is preferable. It is a surprise to me that ether should have so universally superceded the use of chloroform in nearly all surgical operations, whether major or minor. Should a patient die on our hands from the administration of chloroform, then would we become an advocate for the use of ether, but not till then. It is not exaggeration for us to say that we have administered chloroform since 1848 to several hundred individuals of both sexes, and of all ages, with no unhappy results thus far. We have, therefore, a personal experience which induces us to continue the use of the anæsthetic from which we have derived good results and experienced no bad results. I desire, however, to be well understood, and will say here, that I do not regard chloroform safe nor unsafe in all instances.

A patient of ours, in labor, made not to exceed three inspirations before she became entirely insensible and unconscious from inhaling the vapor from a napkin on which had been poured about one drachm of chloroform. On her restoration to consciousness, which occupied perhaps two minutes of times, the chloroform was repeated. I was admonished to extreme caution on my next trial, but the napkin had scarcely been put within inhaling distance, or scarcely a single inspiration had been made before my patient was soundly asleep; from that moment I desisted from further use of the anæsthetic, regarding it as extremely hazardous to make even another trial. I never had any doubt that had carelessness been exercised in its administration in this instance that one more case would have been added to the record of deaths from chloroform.

One person is much more susceptible than another to its power, and the same person is not equally susceptible at all times; hence great caution and persistent vigilance should be exercised always. Chloroform will enjoy a greater reputation for uniformity in its effects upon different persons, if its use be immediately preceded by one or two ounces of brandy or other stimulus, and therefore as a logical sequence its unsafeness will be averted, or if not averted, will be in the exact ratio to its uniformity of action.

When we become mindful that the operation for vesico-vaginal fistula is a long and difficult one, that the patient need not be continuously anæsthetized, that chloroform acts so much more rapidly than ether, and that the position of the patient is a peculiar position, it seems almost incredible to us that any surgeon would, from choice, in any case involving this particular operation, select ether and discard chloroform.

While upon the subject of the particular anæsthesia most desirable for use in the operation under consideration, it will not be inappropriate, I trust, to our present purpose, and to the subject matter under discussion, if we extend our remarks somewhat and make a brief exploration into a hitherto uncultivated field—not to write up the history of the rise, progress and possible decline of chloroform, but rather with a view to see if there may not have been at work in certain quarters an influence prejudicial to its use, an influence so potent that the advocates of chloroform have been obliged to bow before it. But before proceeding with argument in this direction, I desire to pause for a moment to say that if any thing herein be written that might be distorted or misunderstood as an expression or implication that the writer charges any fatal case from use of chloroform to carelessness in its administration, that he herewith disclaims any such intention or purpose.

Many persons may have recollection of the celebrated correspondence upon the subject of the use of chloroform during the years 1848–9, between Drs. Simpson and Meigs. It was the good fortune of the writer to have been a pupil, though not a disciple, of Dr. Meigs at that time. Dr. Simpson was using chloroform freely in his own practice in his own country, and was endeavoring to gain consent of, and induce the great obstetrician of this country to use it here. While some of these letters of Simpson were noted for elegance of diction, were unexceptionable in tone and style, and logical in argument, others degenerated into sarcasm and invective, and some of the rejoinders of Meigs were written with a pen dipped in fluid strongly tinctured with the bitterness of gall and wormwood.

I remember being present on one occasion when Meigs destroyed the life of a rooster in one minute, and of a sheep in seven minutes, from the inhalation of the vapor, but I observed that he allowed no intermixture of air with the vapor. Turning to his pupils Dr. Meigs exclaimed, "that an agent capable of destroying life almost instantaneously, as he had then demonstrated, was a dangerous agent, and should not be used, and that he should never consent to its use, especially in the practice of midwifery, that if he should be instrumental in causing the death of one individual in a thousand to whom he had administered it, that he would cover himself in sackcloth and cast ashes upon his head and bewail his great misfortune and folly during the balance of his days."

Now, therefore, I simply make the suggestion that the burning words of this great and good man may have had their effect upon his countrymen in turning them away from the use of the more speedy and powerful to the more tardy, inert and safe anæsthetic. Five hundred medical students gave audience to the teachings and adopted the opinions of this learned and enthusiastic Professor. In ten years five thousand students would become converted to his views, and when they disperse and are distributed over a con siderable area of territory, occupying different locations in the cities, villages and rural districts of our country their influence is potent for or against truth or error.

Again, the competition and rivalry between medical colleges and hospitals, have had their influence for or against the use of one or other of the anæsthetics. At one time during the history of these agents should you have visited the hospitals of one city you would have found chloroform used. Go to the hospitals of another city and you would have found ether used; one school apparently discarding for no other valid reason than that another school adopted the use of chloroform.

It might not be unprofitable now for a moment to tnrn to the logic of the mooted question. The advocates of ether claim for it immunity from danger and death. Not a single death, say they, has yet been recorded from the use of ether, while on the other hand chloroform has killed its hundreds; therefore chloroform should never be used. Apply this logic to any one of the remedial agents and observe the fallacy of it. Take, if you please, the narcotics—take the class Papaveracie, order papaver somnifirnum and its alkaloids; crude opium is safe as a remedial agent and may even be eaten largely with impunity, it does not cause death. Morphia is more powerful and dangerous; deaths from its use are on record by the hundreds, therefore morphia should not be used.

Space will not permit me and inclination is wanting to pursue this line of argument further, and hence will leave the mooted-question of the relative merits or demerits of the two agents to be decided by our readers.

The best method undoubtedly for the administration of chloroform is that now generally resorted to with the folded napkin, on which is poured two drachms of the fluid, and held sufficiently near the mouth and nostrils to prevent much escape of the vapor and so as to allow as much as possible to enter the respiratory passages with a reasonable intermixture of atmospheric air.—Patients should be directed to inhale and not to swallow the vapor, or rather I should say that patients should be directed to perform voluntary acts of inspiration rather than of deglutition. Such directions will not appear so insignificant and useless when we take into consideration that the large majority of patients seem eager to swallow the vapor, if indeed it could be taken into the stomach, while at first they resist its inhalation or refrain from making the necessary efforts to respire it.

An important desideratum with view to safety, would be to remove the napkin after two or three inspirations and wait to see how it was effecting the patient, then to resume it, and again discoutinue its use for a moment; in this way one may ascertain whether little or much of the vapor will be tolorated, and thereby perhaps avoid all danger.

But it was not our purpose to write a dissertation upon the subject of the use of this agent. We had in the onset designed to discourse upon another subject, but as the opportunity presented itself we have indulged ourselves to this extent, and hope to be forgiven by the readers of the Journal if we have too much digressed from our original purpose, and diverting their minds from the description of an operation tangible to the elucidation of a subject somewhat etherial.

If the readers will take into account that this paper is but a continuation of a former one, it will be conceded that the subject matter herein discussed and the caption on the title page are not, after all, so inappropriate as would appear. If, however, there seems to be in the minds of our readers no connection between the title and body of the paper, I would simply suggest the expediency of substituting chloroform in the heading for vesico-vaginal fistula, and then it will be, in the language of the little Japanese, "all right."

---

CLITORIDECTOMY.—M. Caffee, editor of the "Journal des Connaisances Médicales," states that he assisted M. Michon some time ago in an operation of this kind on the sister of a general. The patient, who was suffering from erotic mania, recovered perfectly and remained free from her malady.

